# Targeting MMP-9 in Diabetic Foot Ulcers

**DOI:** 10.3390/ph12020079

**Published:** 2019-05-22

**Authors:** Jeffrey I. Jones, Trung T. Nguyen, Zhihong Peng, Mayland Chang

**Affiliations:** Department of Chemistry and Biochemistry, University of Notre Dame, Notre Dame, IN 46556, USA; jjones33@nd.edu (J.I.J.); Trung.T.Nguyen.165@nd.edu (T.T.N.); Zhihong.Peng.11@nd.edu (Z.P.)

**Keywords:** matrix metalloproteinase-9, diabetic foot ulcers, wound healing, MMP-9 inhibitors

## Abstract

Diabetic foot ulcers (DFUs) are significant complications of diabetes and an unmet medical need. Matrix metalloproteinases (MMPs) play important roles in the pathology of wounds and in the wound healing process. However, because of the challenge in distinguishing active MMPs from the two catalytically inactive forms of MMPs and the clinical failure of broad-spectrum MMP inhibitors in cancer, MMPs have not been a target for treatment of DFUs until recently. This review covers the discovery of active MMP-9 as the biochemical culprit in the recalcitrance of diabetic wounds to healing and targeting this proteinase as a novel approach for the treatment of DFUs. Active MMP-8 and MMP-9 were observed in mouse and human diabetic wounds using a batimastat affinity resin and proteomics. MMP-9 was shown to play a detrimental role in diabetic wound healing, whereas MMP-8 was beneficial. A new class of selective MMP-9 inhibitors shows clinical promise for the treatment of DFUs.

## 1. Introduction

The skin, one of the largest human organs, is responsible for three critical roles: protection from the environment, maintaining homeostasis, and sensing the surroundings. When the skin is damaged, it undergoes a process termed wound healing. This is a progression of orderly and overlapping processes that culminates in repairing damage to the skin. The four stages of wound healing are inflammation, angiogenesis, re-epithelialization, and remodeling [1]. In an acute wound, there is a smooth and orderly progression through these stages, resulting in healing in a timely manner. Chronic wounds, in contrast, stall in the inflammation stage and do not heal [2]. Stress can adversely affect wound healing [3]. Cortisol, a steroid hormone, is elevated during stress, as well as in chronic inflammation [4], and delays wound healing [5]. CYP11B1, the enzyme that catalyzes biosynthesis of cortisol [6], has been found to be strongly linked to failure of diabetic foot ulcer (DFU) to heal [7]. Elevated MMP-9 levels have been associated with increased cortisol in patients with coronary artery disease [8]. Prostaglandin E2 is known to stimulate cortisol secretion and induce MMP-9 [9]. One of the culprits in the recalcitrance is the elevated activity of matrix metalloproteinases (MMPs) [10].

**Figure 1:**
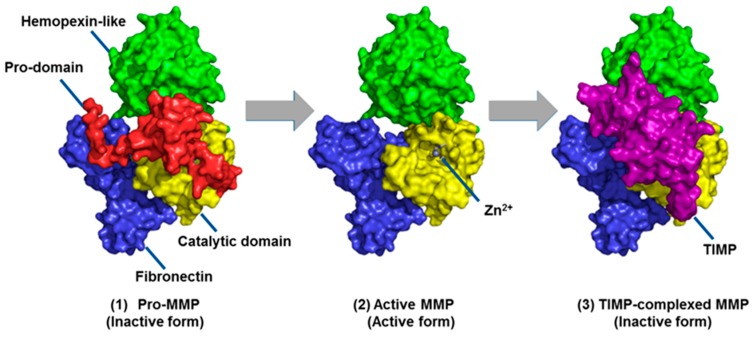
Three forms of matrix metalloproteinases (MMPs), as demonstrated for MMP-2: inactive zymogen (pro-MMP), active MMP, and inactive tissue inhibitor of metalloproteinases (TIMP)-complexed MMP. Cleavage of the prodomain (red) allows access to the catalytic site, while TIMPs block access to inactivate the MMP. Reproduced from Nguyen et al. [13].

MMPs are a family of zinc-dependent endopeptidases first discovered in tadpoles in 1960 [11]. There are 24 different MMPs in humans, with a wide range of substrates and functions [12]. One of the ways MMPs are classified is based on their preferred substrates, such as the gelatinases (MMP-2 and MMP-9), collagenases (MMP-1, MMP-8, and MMP-13), and stromelysins (MMP-3 and MMP-10). Due to the high structural similarities among MMPs, these enzymes share significant overlap in their substrate preferences. All MMPs are produced in inactive zymogen form that requires cleavage of the prodomain to expose the active site [13,14] (Figure 1). Cleavage to the active form by proteases, other MMPs, and post-translational modifications, and allosterically induced self-cleavage, is a secondary method of regulation alongside transcription for MMPs [15,16,17]. MMP activity is further regulated by inhibition of the active forms by tissue inhibitors of metalloproteinases (TIMPs), resulting in three forms of MMPs in vivo (Figure 1), of which only the active, uncomplexed MMP is catalytically competent [18].

Due to the limitations of these methods in studying MMPs, the identification of active MMPs involved in the pathology of cancers was not possible decades ago. MMP inhibitors (MMPIs) were rushed to clinical trials in cancer, based on overexpression of MMPs in cancer cell lines [19] and the protective effects observed with overexpression of TIMPs [20]. The first-generation MMPIs were broad-spectrum zinc chelators with poor bioavailability, which led to a second generation of orally bioactive broad-spectrum inhibitors [21]. However, indiscriminate inhibition of all MMPs resulted in undesirable side effects, such as musculoskeletal pain and inflammation observed during phase I clinical studies [22]. While these proved to be reversible with brief drug holidays, it led to limitations on dose size in subsequent trials [21]. Combination phase II/III studies had disappointing results as well, ranging from no effect to decreased survival [23]. Overall, more than 50 MMPIs failed in clinical trials for cancer [24]. It is now believed that nonspecific chelation and other off-target effects were responsible for these failures [22,25], as well as the lack of understanding that some MMPs can play a beneficial role in cancer [26]. As negative results continued to mount, clinical trials of MMPIs came to a halt, with only one being licensed in the United States, Periostat^®^ (doxycycline), a weak broad-spectrum MMPI, for treatment of periodontal disease [27]. However, MMPs have been found to be dysregulated in a wide variety of diseases beyond cancer, such as stroke [28,29,30] and chronic wounds [31,32,33], which present a new avenue for MMPIs to move to the clinic. This review will focus on DFUs, one of the most common forms of chronic wounds.

Chronic wounds are defined as those that fail to go through the healing process in a timely manner, and are a significant burden on the healthcare system, costing an estimated 2–3% of the healthcare budget in developed countries [34]. DFUs alone cost an estimated $9–13 billion annually in the United States [35]. However, despite this major expenditure, there is a lack of treatment options, with debridement, offloading, and infection control being the standard-of-care. Assessment of wound severity in DFUs is made using the Wagner grade system, ranging from 1, indicating a superficial ulcer, to 5, denoting a complete foot gangrene [36]. In cases that progress to grades 4 and 5, the recourse is sadly amputation, which has significant effects on the quality of life and also is associated with high mortality, 44% after one year [34,37]. Chronic wounds typically stall in the inflammation stage of wound healing, where it is believed that excessive MMP activity plays a role in preventing the wound from healing [38,39].

Many MMPs have been identified in chronic wounds: MMP-1, MMP-2, MMP-3, MMP-7, MMP-8, MMP-9, MMP-10, MMP-12, MMP-13, and MMP-14 [31,40]. However, these MMPs were identified using methods that do not distinguish among the three forms of MMPs. Only active MMPs in the absence of regulation by TIMPs are catalytically competent to play roles in the pathology of chronic wounds. In addition, not all identified MMPs play a deleterious role in the process of wound healing, as shown by treatment with the broad-spectrum inhibitor, GM 6001, which slows healing of human wounds [41]. Given the failure of MMPIs for treatment of cancer, it is important to have a solid understanding of what roles are played by which active unregulated MMP(s).

## 2. Effect of Treatments on MMPs in DFUs

Treatments for DFUs that do not specifically target MMPs can have important effects on MMP expression and activity. As MMP-9 has long been of interest in wound healing, most studies have focused on the effects on this proteinase in particular [42]. These effects are summarized in Table 1.

### 2.1. Vacuum-Assisted Closure

A treatment option that has drawn attention since 1993 is vacuum-assisted closure (VAC), also known as topical negative pressure [50]. VAC involves placing foam around the wound, covering with a soft dressing, and then vacuum is applied to the covered wound via drainage tubes. Multiple studies have shown that VAC promotes wound healing in chronic wounds [50,51,52]. A small-scale study showed a reduction in MMP-9 and MMP-2 by gelatin zymography in chronic wounds of human patients treated with VAC, though the small number of subjects precludes any meaningful statistics [43]. This sparked interest in a larger-scale follow-up study, where it was observed that the increased MMP-9/TIMP-1 ratio in conventionally treated patients is not seen in patients treated with VAC. While there was no significant change in the reported MMP-9 over the course of the study, the use of an ELISA-based assay in addition to sample handling concerns support the argument that this is an artifact of the study [44].

### 2.2. Mesenchymal Stem Cells

Mesenchymal stem cells (MSCs), also known as mesenchymal stromal cells, are a promising strategy for promoting wound healing [53,54,55]. MSCs can be isolated from a number of sources, most importantly bone marrow and adipose tissue, the latter of which allows for easy collection with minimal discomfort [56]. Once isolated, the MSCs are re-injected back into the wound area and have been reported to significantly improve healing in a mouse model of diabetic ulcers [57]. Another method of treatment is to impregnate a hydrogel with MSCs, an approach that shows efficacy in a mouse model of diabetic wounds [58]. Recently, treatment with MSCs in a diabetic animal model was shown to significantly reduce MMP-9 expression by gelatin zymography, and upregulate microRNA miR-29b, a known regulator for MMP-9 [45].

### 2.3. N-Acetyl Cysteine

Another possible treatment to help improve healing in chronic wounds is *N*-acetyl cysteine (NAC), which has a long-track history of safe usage in a variety of diseases, ranging from chronic bronchitis to premature birth [59]. NAC has a strong anti-oxidant effect, which helps fight inflammation and combat oxidative stress [60]. In particular, it is believed to prevent the formation of advanced glycosylation end products (AGE) in diabetics [46]. There is mounting evidence that suggests AGEs are significant contributors to diabetic complications [61]. As a result, NAC has been shown to improve healing in a mouse model of diabetic wounds [62]. Methylglyoxal (MGO) is a key intermediate in AGE production [63]. Treating HaCaT cells with MGO, mimicking the condition found in diabetic tissue, induces MMP-9 expression. However, there was a significant reduction in the expression of MMP-9 by Western blot in cells treated with NAC [46].

### 2.4. Wound Dressings

Advanced wound dressings are another possible method to help improve wound healing by combating infection given the rise of multidrug-resistant bacteria. A study looking at the effectiveness of nano-silver and Manuka honey wound dressings compared to traditional dressings in human DFUs demonstrated a small but significant reduction in ulcer size for the nano-silver dressing. The Manuka honey group did not show a significant improvement in healing, which might be explained by the dramatic increase in MMP-9 observed by ELISA, even relative to the control group [47].

### 2.5. Growth Factors

Growth factors have been an area of considerable focus for drug development to treat DFUs, with at least 28 clinical trials conducted as of 2015. Meta-analysis of these trials found no clear evidence that treatment with growth factors can reduce the number of lower-limb amputations in DFUs [64]. Although human growth factors improve outcomes in complete wound closure, no benefits could be shown in the rate of adverse effects, in addition to concerns about bias in study design and reporting [64]. The sole Federal Drug Administration (FDA)-approved drug for treatment of DFUs is becaplermin, but a “black-box warning” was added in 2008 due to an increased rate of cancer and mortality [65], which was removed in January of 2019 after the manufacturer cited multiple studies that showed no increased safety risk. Becaplermin contains human-platelet-derived growth factor (PDGF)-BB, whereas PDGF-D has been shown to upregulate the expression of TIMP-1, which decreases MMP-2 and MMP-9 activity [66]. Recent results show that treatment of diabetic mouse wounds with becaplermim does improve healing compared to vehicle [48]. In addition, it was observed by in-situ zymography that there is a decrease in the activity of gelatinases, but no effect on collagenases [48], although dye-quenched (DQ)-gelatin is digested by gelatinases and collagenases. DQ-collagen type I fluorescein conjugate (D-12060) can be digested by MMP-1, MMP-2, and MMP-8; mice do not express MMP-1, and MMP-2 is not present in mouse diabetic wounds [67]. However, use of a batimastat affinity resin that binds only to the active forms of MMPs, followed by mass spectrometry (MS)/MS analysis, demonstrated that becaplermin decreases active MMP-9 and does not affect active MMP-8 levels [48].

Aclerastide, also known as NorLeu^3^-A(1–7), is a peptide analog of angiotensin II, a human growth factor [68]. Angiotensin II is known to have an important role in wound healing [69,70], and aclerastide has been reported to improve healing in diabetic mice when administered topically immediately after wound infliction once a day for 5 days [71]. A phase II clinical trial involving 77 patients receiving placebo or aclerastide showed differences in healing and time to ulcer healing in the per-protocol group (*p* < 0.05), but not in the intend-to-treat population (*p* > 0.05) [72]. However, aclerastide failed phase III clinical trials in 2015 due to a lack of efficacy after topical administration once a day for 28 days. Recent research using a more clinically relevant dosing regimen (topical administration once a day for 14 days starting one day after wound infliction) showed that aclerastide did not accelerate wound healing in diabetic mice (*p* > 0.05) and that the lack of efficacy in human clinical trials is likely due to upregulation of active MMP-9 [49], as determined by a batimastat affinity resin coupled with proteomics. Previously, angiotensin II had been shown to increase the mRNA expression of MMP-9 by reverse transcription polymerase chain reaction (RT-PCR), as well as the protein expression by Western blotting [73], a method that does not distinguish between the three forms of MMP-9. 

## 3. Affinity Enrichment Approaches to Identify MMPs

As evidence for a deleterious role of MMP-9 in wound healing and its correlation with wound healing increased, MMP-9 has been suspected of having a causal role in the delayed healing of DFUs [2,42,74,75]. However, the role of MMP-9 in DFUs had not been conclusively determined because the methods used do not distinguish between the three forms of MMP-9, of which only active MMP-9 in the absence of regulation by complexation with TIMP is catalytically competent to play a role in the pathology of DFUs.

Homology between the three forms of MMPs presents a significant analytical challenge in measuring only the active MMPs [76,77]. Activity-based techniques, such as gelatin zymography, are semi-quantitative at best and cannot distinguish between the TIMP-inhibited and the active form of the proteinase due to dissociation of TIMP during analysis [76]. Measurements of expression via mRNA, made using RT-PCR, do not give information about the activation from the zymogen form nor inactivation via TIMPs. Antibody-based assays, such as ELISA or Western blots, use antibodies that are not necessarily specific to the active form; thus, there is cross-reactivity between pro-MMPs, active MMPs, and TIMP-complexed MMPs [76]. An additional drawback of these methods is that they require screening for a specific MMP rather than simultaneously identifying the MMP(s) that plays critical roles in the pathology and repair of DFUs. MS, the gold-standard method for protein quantitation [78], cannot differentiate between the three forms in a standard bottom-up proteomics experiment. A conventional proteomics strategy separates proteins in a biological sample extract by 1D/2D high performance liquid chromatography (HPLC) and identifies the trypsin-digested peptides by mass spectrometry (MS)/MS. This strategy identifies thousands of proteins.

In order to enrich the proteinases, an MMPI has been covalently attached to a resin, allowing for isolation of the active MMP forms alone for identification and quantitation. Initially, a bifunctional probe HxBP-Rh based on the structure of the broad-spectrum MMPI illomastat was synthesized, which contained a fluorescent rhodamine group and a photoreactive benzophenone for covalent binding to the target MMPs [79]. HxBP-Rh was demonstrated to bind active MMP-2, MMP-7, and MMP-9. To enable affinity purification, a trifunctional probe of HxBP-Rx was synthesized incorporating biotin. Application of this method coupled to MS/MS identified the metalloendopeptidase neprilysin in invasive melanoma [79].

**Figure 2:**
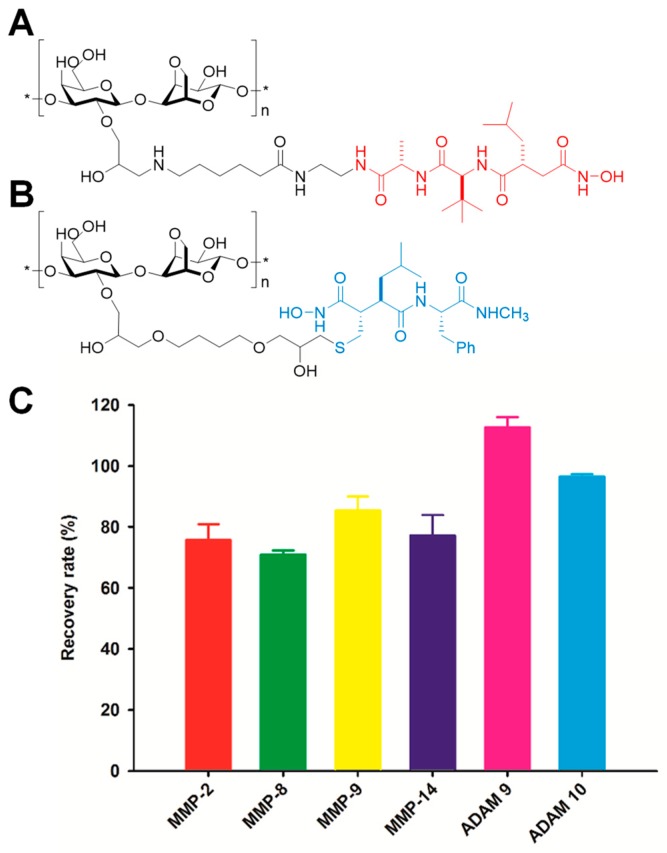
Structures of (**A**) the TAPI-2 affinity resin and (**B**) the batimastat affinity resin used to capture active MMPs and related ADAMs (a disintegrin and metalloproteinase). The portion of the structure based on TAPI-2 is indicated in red and that based on batimastat is shown in blue. (**C**) Recovery of representative active MMPs and ADAMs by the batimastat affinity resin. Mouse tissue homogenate was spiked with 10 pmol of proteinase and incubated with the resin. Recovery was determined from quantitation of the proteinase recovered after the clean-up procedure relative to the spiked amount; mean ± SD, *n* = 3.

Another approach is the broad-spectrum MMPI TAPI-2 covalently attached to Sepharose resin (Figure 2A) [80,81], which can be packed on a cartridge and proteinases are eluted with EDTA. Recoveries of MMP-1, -7, -8, -9, -10, -12, and -13 were >96% when injected in buffer at a concentration of 0.5 μg/mL. Synovial fluid from a patient with rheumatoid arthritis was analyzed by this method coupled with gelatin zymography, and showed enrichment of MMP-9.

A third approach is the covalent tethering of the broad-spectrum inhibitor batimastat to Sepharose resin (Figure 2B) [81]. The batimastat affinity resin binds exclusively to the active forms of MMPs. As seen in Figure 1, pro-MMPs have the prodomain (depicted in red) covering the active site, while TIMP-complexed MMPs have TIMP (depicted in purple) blocking the active site; only the active MMPs (Figure 1 center) have the active site available for binding of the affinity resin. Recoveries of representative MMPs and ADAMs (a disintegrin and metalloproteinase) are >75% (Figure 2C). The limit of quantitation is 6 femtomol [67]. Application of this method coupled with gelatin zymography showed the presence of active MMP-2 in breast carcinoma samples [81]. 

## 4. The Roles of MMPs in DFUs

The batimastat affinity resin is the only approach that has been applied to the identification of active MMPs in DFUs. Excisional wounds in diabetic mice treated with the batimastat affinity resin coupled with a bottom-up proteomics approach showed upregulated levels of active MMP-8 and MMP-9 compared to non-diabetic mouse wounds [67]. The question was what roles these two MMPs play in DFUs. Answers to these questions require availability of selective MMP-8 and MMP-9 inhibitors. However, because of the structural similarity between MMPs, selective inhibitors for a particular MMP are difficult to design.

The first selective inhibitor for the gelatinases (MMP-2 and MMP-9), referred to as SB-3CT, was reported by Brown et al. [82]. SB-3CT (Table 2) inhibits MMP-2 and MMP-9 with *K_i_* values of 28 ± 7 nM and 400 ± 15 nM, respectively, and inhibits other MMPs poorly [82,83]. The selectivity of SB-3CT towards the gelatinases is attributed to mechanism-based inhibition, in which Glu-404 at the active site of the gelatinases abstracts a proton α to the sulfone, opening the thiirane ring to a thiolate, which is a tight-binding inhibitor that coordinates with zinc ion [84]. This results in residence times (the time the inhibitor is bound to the gelatinases) that are very long, as the reversal of this reaction is slow. SB-3CT inhibits MMP-8 as a non-competitive inhibitor with a *Ki* value of 2100 ± 400 nM [83], for a selectivity towards MMP-9 relative to MMP-8 of 3.5 (Table 2). A drawback of SB-3CT is its poor water solubility of 2.3 μg/mL [85].

**Figure 3:**
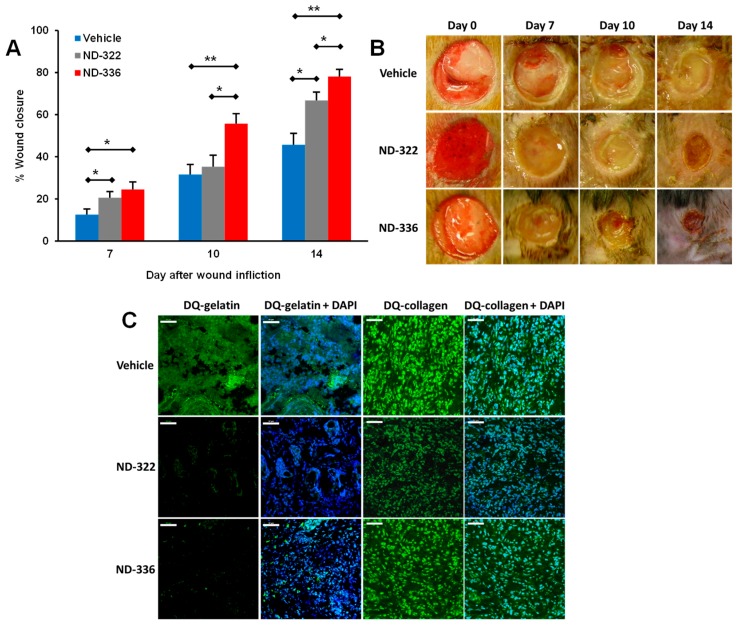
Selective MMP-9 inhibition accelerates diabetic wound healing. (**A**) Wound healing after treatment with 100 μg/wound/day of (*R,S*)-ND-336, (*R,S*)-ND-322, or vehicle. Mean ± standard error of the mean (SEM); *n* = 8/group on days 7, 10, and 14; * *p* < 0.05, ** *p* < 0.01 by Mann–Whitney *U* test. (**B**) Representative images of the wounds. (**C**) In-situ zymography of wounds with DQ-Gelatin and DQ-Collagen indicates that (*R,S*)-ND-336 inhibits MMP-9 but not MMP-8, while (*R,S*)-ND-322 inhibits MMP-9 and poorly inhibits MMP-8. Reproduced from Gao et al [86].

In search of water-soluble selective MMP-9 inhibitors, (*R,S*)-ND-322 was discovered with a water solubility of 4.9 mg/mL [87], inhibiting MMP-9 in a slow-binding manner with a *K*_i_ of 870 ± 110 nM and inhibiting MMP-8 as a non-competitive inhibitor with a *K*_i_ of 2600 ± 400 nM (Table 2) [67]. Topical administration of (*R,S*)-ND-322 accelerated wound healing in diabetic mice [67]. However, (*R,S*)-ND-322 is metabolized by *N*-acetylation [87]. Replacement of the aniline with methylaminophenyl to block metabolism by *N*-acetylation resulted in (*R,S*)-ND-336, which retained water solubility of 4.9 mg/mL and with increased potency (*K*_i_ of 150 ± 10 nM for MMP-9 and 7700 ± 100 nM for MMP-8) and selectivity (Table 2) [86]. (*R,S*)-ND-336 showed better efficacy in accelerating diabetic wound healing than (*R,S*)-ND-322 (Figure 3a,b), which was attributed to improved selectivity towards inhibition of MMP-9 relative to MMP-8 (Figure 3c) [86].

**Figure 4:**
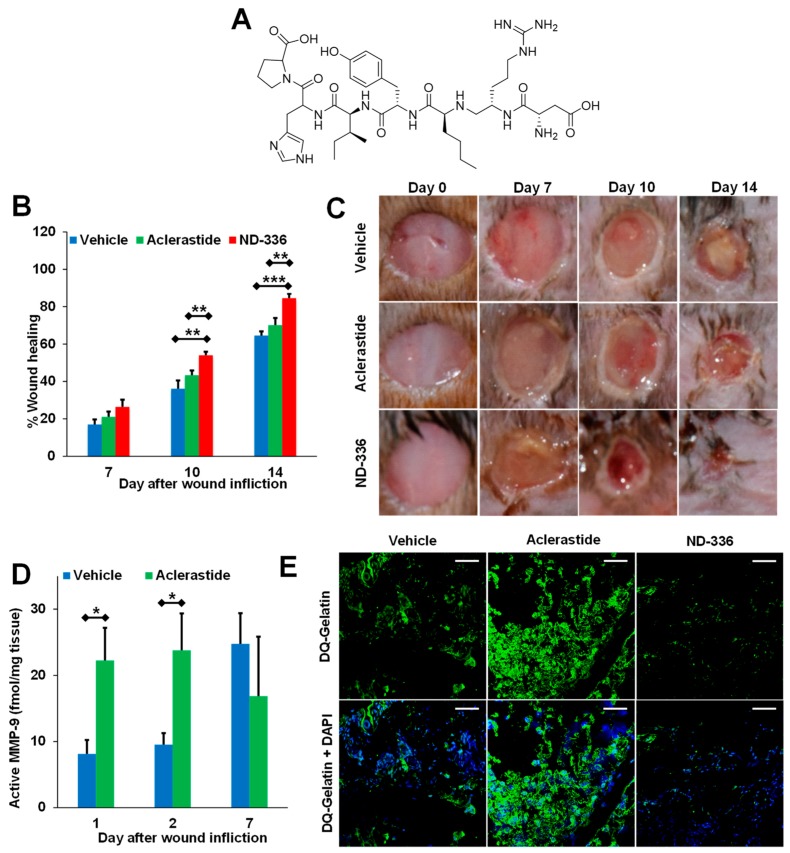
Aclerastide does not accelerate wound healing in diabetic mice when given at a clinically relevant dosing regimen. (**A**) Structure of aclerastide. Mice were treated topically 1 day after wound infliction with (*R,S*)-ND-336 or aclerastide at 100 µg/wound/day for 14 days or vehicle (water). (**B**) Wound closure measurements indicate that (*R,S*)-ND-336 accelerates wound healing faster than aclerastide; mean ± SEM, *n* = 12, 9, and 9 mice for (*R,S*)-ND-336 or aclerastide on days 7, 10, and 14, respectively; *n* = 13, 10, and 10 mice for vehicle on days 7, 10, and 14, respectively; ** *p* < 0.01, *** *p* < 0.001 by Mann–Whitney *U* two-tailed test. (**C**) Representative images of the wounds. (**D**) Increased active MMP-9 levels are observed in aclerastide-treated wounds by the batimastat affinity resin coupled with proteomics; mean ± S.D., *n* = 3 mice per group per time point, * *p* < 0.05 by Student’s t two-tailed test. (**E**) Aclerastide treatment increases the detrimental MMP-9 activity in diabetic wounds by in-situ zymography with DQ-Gelatin, whereas MMP-9 activity was inhibited in (*R,S*)-ND-336-treated wounds. Adapted from Nguyen et al. [49].

The detrimental role of MMP-9 was confirmed with MMP-9 knockout mice treated with streptozotocin in order to induce diabetes. The diabetic MMP-9 knockout animals showed improved healing compared to streptozotocin-treated wild-type mice [86].

(*R,S*)-ND-336 was shown to have better efficacy than aclerastide (Figure 4A–C) [49], an analog of angiotensin II that failed in phase III clinical trials for the treatment of DFUs. Aclerastide-treated wounds resulted in increased levels of active MMP-9 as demonstrated by with the batimastat affinity resin/proteomics method (Figure 4D) and in-situ zymography (Figure 4E). Aclerastide had previously shown efficacy in diabetic mice when administered immediately after wound infliction and when given for a shorter dosing regimen [71]. Aclerastide’s beneficial effect in promoting wound healing by inducing angiogenesis and migration of fibroblasts and keratinocytes [69,88] and its detrimental effect in delaying wound healing by increasing active MMP-9 cancelled each other, resulting in wound healing similar to that in vehicle-treated mice. This study found that expression of active MMP-9 likely contributed to aclerastide’s clinical failure in the treatment of DFUs [49].

**Figure 5:**
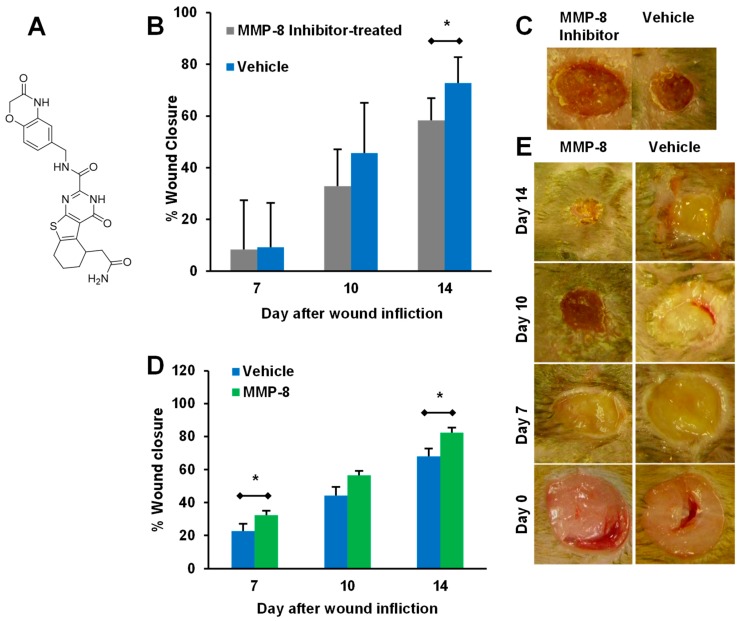
The role of MMP-8 in diabetic wound healing. (**A**) Structure of selective MMP-8 inhibitor. (**B**,**C**) Topical treatment with selective MMP-8 inhibitor (250 μg/wound/day) delays healing in diabetic mice; mean ± SD, *n* = 12, 6, and 6 on days 7, 10, and 14, respectively; * *p* < 0.05. (**D**,**E**) Topical treatment with exogenous recombinant MMP-8 (1 μg/wound/day) accelerates diabetic wound healing; mean ± SEM, *n* = 20, 9, and 9 mice on days 7, 10, and 14, respectively, for the vehicle group; *n* = 20, 10, and 10 mice on days 7, 10, and 14, respectively, for the MMP-8 group; * *p* < 0.05. Adapted from Gooyit et al. [67] and Gao et al. [86].

On the other hand, MMP-8 was shown to be beneficial to diabetic wound healing. Selective chemical inhibition of MMP-8 (Figure 5A) delayed wound healing in diabetic mice (Figure 5B,C) [67]. Confirmation of the beneficial role of MMP-8 was not feasible with MMP-8 knockout mice, as ablation of the MMP-8 gene results in significantly increased levels of MMP-9 [89]. Instead, diabetic mice were treated topically with recombinant MMP-8, which resulted in increased wound healing (Figure 5D,E) [86]. Combination therapy with a selective MMP-9 inhibitor and topical application of MMP-8 further improved healing beyond either of the two individual treatments [86]. 

**Figure 6:**
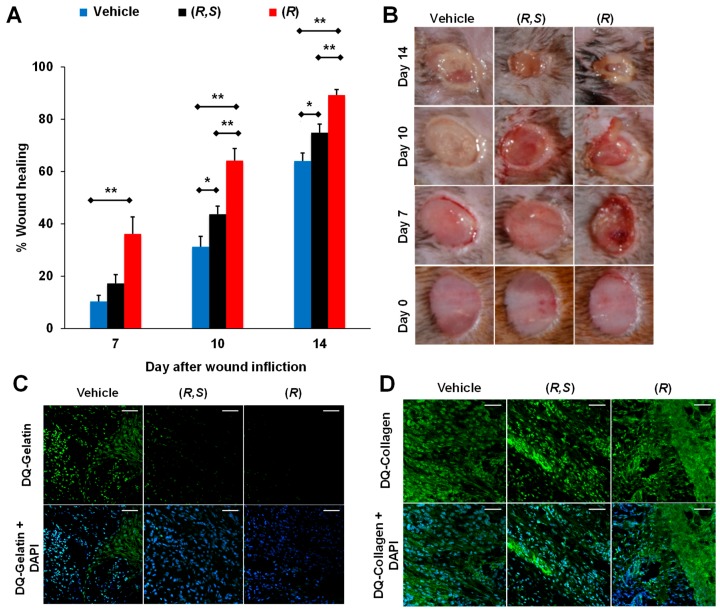
(*R*)-ND-336 accelerates wound healing faster than (*R,S*)-ND-336 in diabetic mice. Topical treatment with (*R*)-, or (*R,S*)-ND-336 at 50 μg/wound/day for 14 days or vehicle (water). (**A**) Wound healing; mean ± SEM, *n* = 7 mice/group/time point for vehicle, (*R,S*)-ND-336, *n* = 8/time point for (*R*)-ND-336, * *p* < 0.05, ** *p* < 0.01 by Mann−Whitney U two-tailed test. (**B**) Representative wound images. (**C**) In-situ zymography of the wounds with DQ-gelatin shows that (*R*)-ND-336 inhibits MMP-9 better than (*R,S*)-ND-336; *n* = 3 mice/group. The bottom row shows merged images with DAPI nuclear DNA staining, 40× lens (scale bars, 50 μm). (**D**) In-situ zymography with DQ-collagen indicates that (*R,S*)- and (*R*)-ND-336 do not inhibit MMP-8; *n* = 3 mice/group. Adapted from Nguyen et al. [48].

Given that MMP-9 is detrimental and MMP-8 is beneficial to healing of DFUs, the best strategy is to selectively inhibit MMP-9 without affecting MMP-8. To this effect, a search for more selective MMP-9 inhibitors led to the discovery of (*R*)-ND-336, which inhibits MMP-9 as a slow-binding inhibitor with a *K*_i_ of 19 ± 3 nM and inhibits MMP-8 as a non-competitive inhibitor with a *K*_i_ of 8590 ± 230 nM (Table 2) [48]. (*R*)-ND-336 had superior efficacy compared to (*R,S*)-ND-336 (Figure 6A,B) due to more potent and selective inhibition of MMP-9 (Figure 6C,D).

**Figure 7:**
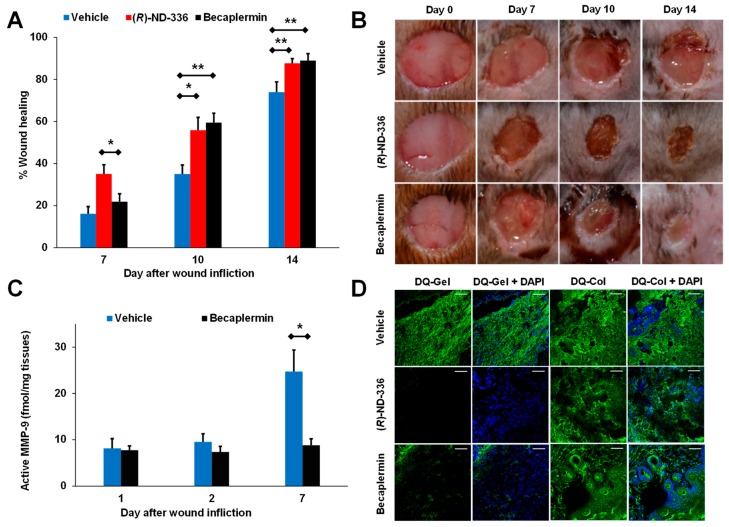
(*R*)-ND-336 is superior to becaplermin in accelerating wound healing in diabetic mice. Mice were treated topically 1 day after wound infliction (8 mm, Tegaderm-covered) with 5 μg or 50 μg/wound/day of becaplermin or (*R*)-ND-336, respectively, or vehicle (water) for 14 days. (**A**) Wound measurements show that (*R*)-ND-336 has better efficacy than becaplermin, mean ± SEM; *n* = 11, 8, and 8 for vehicle, *n* = 11, 7, and 7 for (*R*)-ND-336, *n* = 12, 9, and 9 for becaplermin on days 7, 10, and 14, respectively; * *p* < 0.05, ** *p* < 0.01 by Mann−Whitney U two-tailed test. (**B**) Representative wound images. (**C**) Analysis of the wounds with the affinity resin coupled to proteomics indicates significant decrease in active MMP-9 in becaplermin-treated animals. (**D**) In-situ zymography with DQ-gelatin shows that (*R*)-ND-336 inhibits MMP-9 activity in vivo, while becaplermin decreases MMP-9 activity but does not completely inhibit it. In-situ zymography with DQ-collagen shows that (*R*)-ND-336 and becaplermin do not inhibit MMP-8. Images were taken with a 40× lens (scale bars, 50 μm). Adapted from Nguyen et al. [48].

Becaplermin remains the only FDA-approved drug for the treatment of DFUs since it was approved in 1997. A side-by-side comparison of (*R*)-ND-336 and becaplermin demonstrated that the former had superior efficacy early after treatment (Figure 7A,B), while becaplermin accelerated wound healing relative to diabetic mice treated with vehicle at later days, paralleling what has been observed in clinical trials in which becaplermin differentiates from placebo in complete wound healing incidence after 10-week treatment [90]. The superior efficacy of (*R*)-ND-336 was attributed to complete inhibition of MMP-9 (Figure 7C) [48]. Becaplermin poorly inhibits MMP-9 (17% inhibition at 4 μM) and indirectly decreases MMP-9 activity, but does not completely inhibit it. This was confirmed with the batimastat affinity resin, which resulted in 3-fold decrease in active MMP-9 levels compared to vehicle control on day 7 (Figure 7C,D) [48]. This study showed that becaplermin reduces active MMP-9, and this may explain its efficacy.

**Figure 8:**
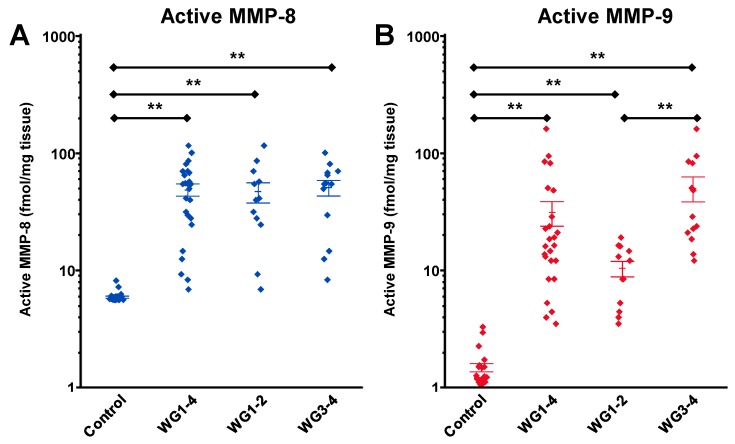
Validation of the target MMP-9 in debridement tissue from human patients with diabetic foot ulcers (DFUs). Measurements of (**A**) active MMP-8 and (**B**) active MMP-9 in human DFUs stratified by the Wagner grade (WG) using the batimastat affinity resin coupled with proteomics; mean ± SEM: * *p* < 0.05, ** *p* < 0.01 by Mann−Whitney U two-tailed test. Adapted from Nguyen at al. [48].

These studies conclusively determined that MMP-9 has a detrimental role in healing of DFUs and that MMP-8 has a beneficial one. This is further supported by validation of the target MMP-9 in debridement tissue from humans with DFUs, where DFUs were stratified by the Wagner grade (WG) of the ulcer [48]. MMP-8 showed no correlation with ulcer grade (Figure 8A), while WG3-4 ulcers have significantly higher concentrations of active MMP-9 than WG1-2 (Figure 8B), which in turn were higher than control [48].

Upon injury, neutrophil levels increase at the site to help fight infection. MMP-8, MMP-9, and reactive oxygen species (ROS) are secreted by neutrophils. ROS function to kill bacteria, as well as help in formation of a thrombus that protects the wound preventing further blood loss. ROS activates NF-кB, which upregulates MMP-9 and is detrimental to DFU healing [48]. On the other hand, the function of MMP-8 is to repair damaged collagen and in re-epithelialization that is critical for the wound healing process [89].

## 5. Targeting MMP-9 with Therapeutics

Selective inhibition of MMP-9 (gelatinase B) is a challenge because of the shared substrate specificity and homology of MMPs [91,92], especially with MMP-2 (gelatinase A). Even in the face of this challenge, the failure of the early broad-spectrum MMPIs demonstrated the need for selective MMPIs. The confirmed beneficial role of MMP-8 makes selective inhibition of MMP-9 all the more important for the development of a therapeutic for the treatment of DFUs. However, as active MMP-2 is not present in diabetic wounds [67], this is not an insurmountable challenge. There is still a strong effort being made by the community to overcome these challenges and to deliver a selective MMP-9 inhibitor [93].

### 5.1. Small-Molecule Inhibitors

**Figure 9:**
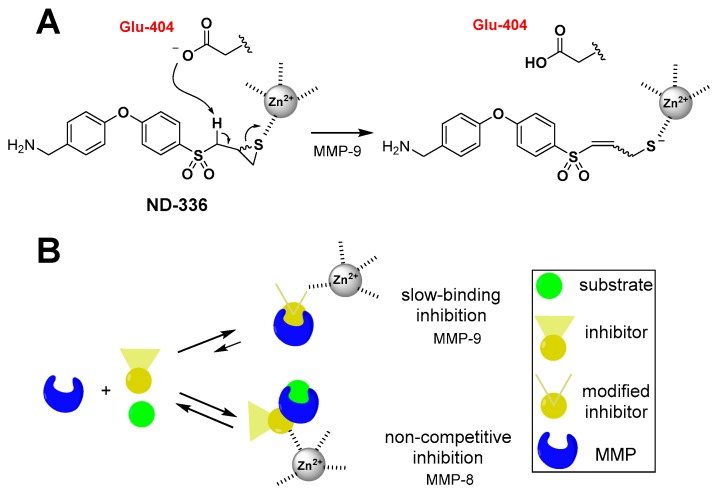
Mechanism of inhibition of (*R*)-ND-336. (**A**) (*R*)-ND-336 inhibits MMP-9 as a mechanism-based inhibitor, where Glu-404 at the active site abstracts a proton α to sulfone, resulting in the corresponding thiolate that coordinates with zinc ion as a tight-binding inhibitor, for which the reversal occurs very slowly. (**B**) (*R*)-ND-336 inhibits MMP-9 as a slow-binding inhibitor with a long residence time of 300 ± 1 min. The compound is a poor non-competitive inhibitor for MMP-8, for which the residence time is very short. Adapted from Nguyen et al. [48].

One of the promising stories for a therapeutic MMPI is the development of (*R*)-ND-336, a member of the thiirane class of MMP-9 inhibitors. Based on the *K*_i_ values, the racemate (*R,S*)-ND-336 has 51-fold selectivity towards MMP-9 compared to MMP-8 [86]. As (*R,S*)-ND-336 accelerated diabetic wound healing in mice, (*R*)- and (*S*)-ND-336 were synthesized as enantiomerically pure isomers. (*R*)-ND-336 was shown to be a 10-fold more potent inhibitor of MMP-9 than (*S*)-ND-336 [48], with 450-fold selectivity towards MMP-9 relative to MMP-8 compared to 11-fold selectivity for (*S*)-ND-336 (Table 2). Selectivity based on *K*_i_ values is further enhanced, as (*R*)-ND-336 inhibits MMP-9 as a mechanism-based inhibitor, undergoing ring-opening at the active site to produce a thiolate that coordinates with the active-site zinc for which the reversal is extremely slow (Figure 9), imparting a long residence time for MMP-9 of 300 ± 1 min and further selectivity when compared to the fast *k*_off_ for MMP-8 (residence time for MMP-8 <1 s) [48]. (*R*)-ND-336 has superior efficacy than becaplermin, the only FDA-approved therapeutic for the treatment of DFUs, indicating its promise as a therapeutic for the treatment of DFUs.

### 5.2. Antibody-Based Inhibitors

One of the possible avenues to address the challenge of selectivity for inhibitors is the use of antibodies. A number of inhibitory antibodies have been developed to target MMPs, such as GS-5745, which targets MMP-9. GS-5745 shows a dual-faceted method of inhibition for MMP-9, sterically hindering both access to the active site (IC_50_ for MMP-9_cat_ = 1.3 ± 0.1 nM) and the ability of the zymogen to be cleaved to the active form by MMP-3 [94]. In addition, it reports being well-tolerated during phase I clinical trials [95,96]. Other antibodies, such as SDS3 (*K*_i_ MMP-9 = 1 µM) [97] and REGA-3G12 [98,99], have been reported as MMP-9 inhibitors. However, all three of these have only been applied toward cancers, and not wound healing. The clinical potential of these inhibitors for wound healing, in the context of the wealth of knowledge about the importance of MMP-9 [42], is a promising but unexplored territory.

### 5.3. Advanced Wound Dressings

As dressings are commonly employed in the clinic to help with wound healing, both to control the wound environment and manage exudate [100], they are an ideal vehicle for altering the MMP profile. There has been interest in tethering an MMPI to a wound dressing, though this currently involves the use of nonspecific inhibitors, such as bisphosphonates [101]. Localization of the inhibitor to the wound site and the use of compounds with a known tolerance has been proposed to minimize the risk of side effects [101]. Another option along this path is a wound dressing composed of type I atelocollagen functionalized with 4-vinylbenzyl chloride, a compound that was hypothesized to complex with active MMPs in wound exudates [102]. Support for this hypothesis was demonstrated by its reduction of MMP-9 activity unlike standard-of-care dressings. As a result, the use of the dressing on a mouse model of diabetic wounds resulted in improved healing when compared to a commercial adhesive control [102]. While this methodology is interesting, the use of a broad-spectrum inhibitor rather than a selective MMP-9 inhibitor is of concern in the context of the reduced wound healing observed with the use of the broad-spectrum MMPI GM 6001 in humans [41].

### 5.4. RNA-Based Therapies

Another option for targeting MMP-9 is to interfere with expression at the gene level. RNA inhibitors are a promising method of treatment for many diseases [103], but face the challenge of delivering the small interfering RNA (siRNA) in a safe and efficient manner [104]. One of the possible tools for delivering the siRNA is cationic star-shaped polymers based on cyclodextrins (CDs) [105,106,107], as they have been shown to have low toxicity [108]. After a study reporting on the overexpression of MMP-9 relative to TIMP-1 in a rat model of diabetic wounds [109], the design and efficacy of β-CD-(D_3_)_7_/MMP-9-siRNA was reported. It was demonstrated to be effectively taken up by fibroblast cells, reducing MMP-9 gene expression, and to significantly improve wound healing in a rat model of diabetic ulcers [110]. A follow-up study looked at toxicity in the liver and kidney, finding only a transient effect for the naked siRNA and none in the β-CD-(D_3_)_7_/MMP-9-siRNA [111]. More recently, there was a report of microRNA(miRNA)-129 and miR-335 as an inhibitor of MMP-9 expression in a diabetic mouse model, which results in a commensurate improvement in wound healing [112].

## 6. Conclusion and Future Perspectives

The initial failure of broad-spectrum MMPIs halted interest in the development of MMPIs as therapeutics. However, as the importance of targeting MMP-9 in the treatment of DFUs has become evident, a generation of selective MMP-9 inhibitors has emerged. There are multiple avenues for selective inhibitor design, from small-molecule inhibitors that selectively target active MMP-9 to RNA inhibitors that aim to block expression of MMP-9 at the gene level.

**Figure 10:**
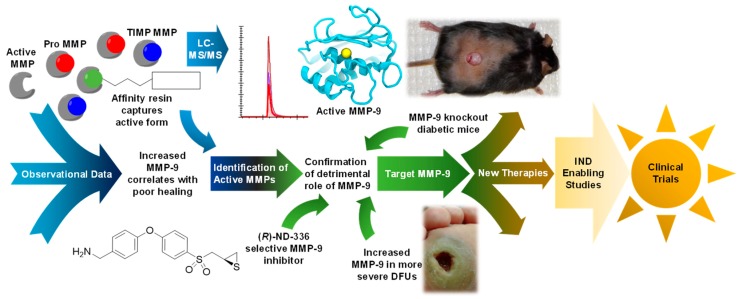
Schematic showing the progress of translational work to bring a selective MMP-9 inhibitor towards the clinic for treatment of DFUs.

As chronic wounds remain a complex challenge, the development of a safe and effective treatment option is of outmost importance. While translation from laboratory to clinic takes time, resources, and effort (Figure 10), there is huge promise shown by the current wave of therapeutics in development.

## Figures and Tables

**Table 1:** Summary of the reported effects on treatments on MMPs and how the MMPs were measured.

Treatment	Study Material	Method of MMP Measuring	Effect on MMPs	Reference
Vacuum-assisted closure (VAC)	Human chronic wound fluid	Gelatin zymography	Reduced MMP-9 and MMP-2	[43]
VAC	Human chronic wound fluid	ELISA	Reduced MMP-9/TIMP-1 ratio, no change in MMP-9	[44]
Mesenchymal stem cells (MSC)	Mouse model of diabetic wounds	Gelatin zymography, quantitative PCR	Reduced MMP-9 activity and expression	[45]
N-acetyl cysteine	HaCat cells treated with MGO	Western blot	Reduced MMP-9 expression	[46]
Manuka honey wound dressing	Human DFU patient wound fluid	ELISA	Increased MMP-9 expression (no improvement on ulcer healing)	[47]
Becaplermin	Mouse model of diabetic wounds	In-situ zymography and batimastat affinity resin coupled proteomics	Decreased gelatinase activity, no effect on collagenase activity; decreased active MMP-9, no effect on active MMP-8	[48]
Aclerastide	Mouse model of diabetic wounds	Batimastat affinity resin coupled proteomics	Increased active MMP-9, no effect on active MMP-8	[49]

**Table 2:** Inhibition constants for the small-molecule inhibitors, showing the progression to selectivity.

*K*_i_ (nM)	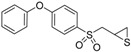	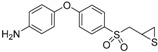	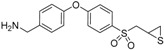	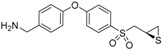
Name	SB-3CT	(*R,S*)-ND-322	(*R,S*)-ND-336	(*R*)-ND-336
MMP-9	400 ± 15	870 ± 110	150 ± 10	19 ± 3
MMP-8	2100 ± 400	2600 ± 400	7700 ± 100	8590 ± 230
Selectivity (*K*_i MMP-8_/*K*_i MMP-9_)	3.5	3.0	51	450
MMP-9 residence time (min)	13.4	31.4	47.4 ± 4.4	300 ± 1
Reference	[82,83]	[67]	[86]	[48]
